# Biochemistry of nicotine metabolism and its relevance to lung cancer

**DOI:** 10.1016/j.jbc.2021.100722

**Published:** 2021-04-29

**Authors:** Sharon E. Murphy

**Affiliations:** Masonic Cancer Center, University of Minnesota, Minneapolis, Minnesota, USA

**Keywords:** cytochrome P450, lung cancer, metabolism, uridine 5′-diphospho-glucuronosyltransferase (UDP-glucuronosyltransferase), cancer, nicotine, cotinine, smoking, CYP2A6, FMO, flavin monooxygenase, NNK, 4-(methylnitrosamino-1-(3-pyridyl)-butanone), P450,CYP, cytochrome P450, UGT, uridine 5′-diphospho-glucuronosyltransferaseglucuronosyltransferase

## Abstract

Nicotine is the key addictive constituent of tobacco. It is not a carcinogen, but it drives smoking and the continued exposure to the many carcinogens present in tobacco. The investigation into nicotine biotransformation has been ongoing for more than 60 years. The dominant pathway of nicotine metabolism in humans is the formation of cotinine, which occurs in two steps. The first step is cytochrome P450 (P450, CYP) 2A6–catalyzed 5′-oxidation to an iminium ion, and the second step is oxidation of the iminium ion to cotinine. The half-life of nicotine is longer in individuals with low P450 2A6 activity, and smokers with low activity often decrease either the intensity of their smoking or the number of cigarettes they use compared with those with “normal” activity. The effect of P450 2A6 activity on smoking may influence one's tobacco-related disease risk. This review provides an overview of nicotine metabolism and a summary of the use of nicotine metabolite biomarkers to define smoking dose. Some more recent findings, for example, the identification of uridine 5′-diphosphoglucuronosyltransferase 2B10 as the catalyst of nicotine *N*-glucuronidation, are discussed. We also describe epidemiology studies that establish the contribution of nicotine metabolism and *CYP2A6* genotype to lung cancer risk, particularly with respect to specific racial/ethnic groups, such as those with Japanese, African, or European ancestry. We conclude that a model of nicotine metabolism and smoking dose could be combined with other lung cancer risk variables to more accurately identify former smokers at the highest risk of lung cancer and to intervene accordingly.

In the United States, an estimated 235,760 new lung cancer cases will be diagnosed, and 131,880 individuals will die from the disease in 2021 (1). Worldwide, lung cancer is the leading cause of cancer death, accounting for 11.6% of the 9.6 million estimated deaths in 2018 (2). Cigarette smoking is the cause of as much as 90% of this cancer (3, 4, 5). However, only 11 to 24% of smokers will develop lung cancer, and for the same reported lifetime quantity of cigarettes, lung cancer risk differs by racial/ethnic group (6, 7). Nicotine, the primary psychoactive compound present in tobacco, is responsible for maintaining smoking behaviors (8), and individual variation in nicotine metabolism is an important contributor to racial/ethnic and individual differences in lung cancer risk (6).

Nicotine is not a carcinogen but is arguably the compound present in tobacco with the greatest influence on a smoker's cancer risk. Nicotine sustains tobacco addiction and continued smoking (9, 10, 11, 12). Upon inhalation, nicotine enters the circulation by way of the lungs. It then travels to the brain where it readily diffuses into the tissue and stereoselectively binds to nicotinic cholinergic receptors. This results in the release of dopamine, which mediates the pleasurable experience of smoking. The time between inhaling a puff of tobacco smoke and the release of dopamine is a few seconds. Each puff contains more than 70 identified carcinogens, many of which contribute to the risk of a smoker developing lung cancer (10, 13). Biological effects of the noncarcinogenic toxicants present in tobacco smoke are also involved. These cocarcinogenic and tumor-promoting compounds contribute to the well-established mechanism of tobacco carcinogenesis (9, 10). However, the entire process is dependent on nicotine. When the nicotine content of a cigarette is reduced below an addictive level, very few individuals continue to smoke these cigarettes (12, 14).

The study of nicotine metabolism began primarily in the purview of chemists, who identified, characterized, and quantified nicotine metabolites in the blood and urine of multiple species. This effort took off in the late 1950s and early 1960s. In 1959, cotinine was identified as the principal nicotine metabolite in the urine of smokers (15). While a hydroxycotinine metabolite was detected in the early study, it was more than 25 years later that *trans* 3′-hydroxycotinine was characterized and found to be the major urinary nicotine metabolite (16). About 5 years after that, cotinine glucuronide was identified and determined to be equally or more abundant than cotinine in a smoker's urine (17, 18). The quantification of cotinine plus these two metabolites led to the realization that cotinine formation by nicotine 5′-oxidation was the critical pathway for the elimination of nicotine in smokers (Fig. 1). Prior to this, the *N*-oxidation of nicotine was believed to be as important or possibly more important than 5′-oxidation in the detoxification of nicotine.

**Figure 1:**
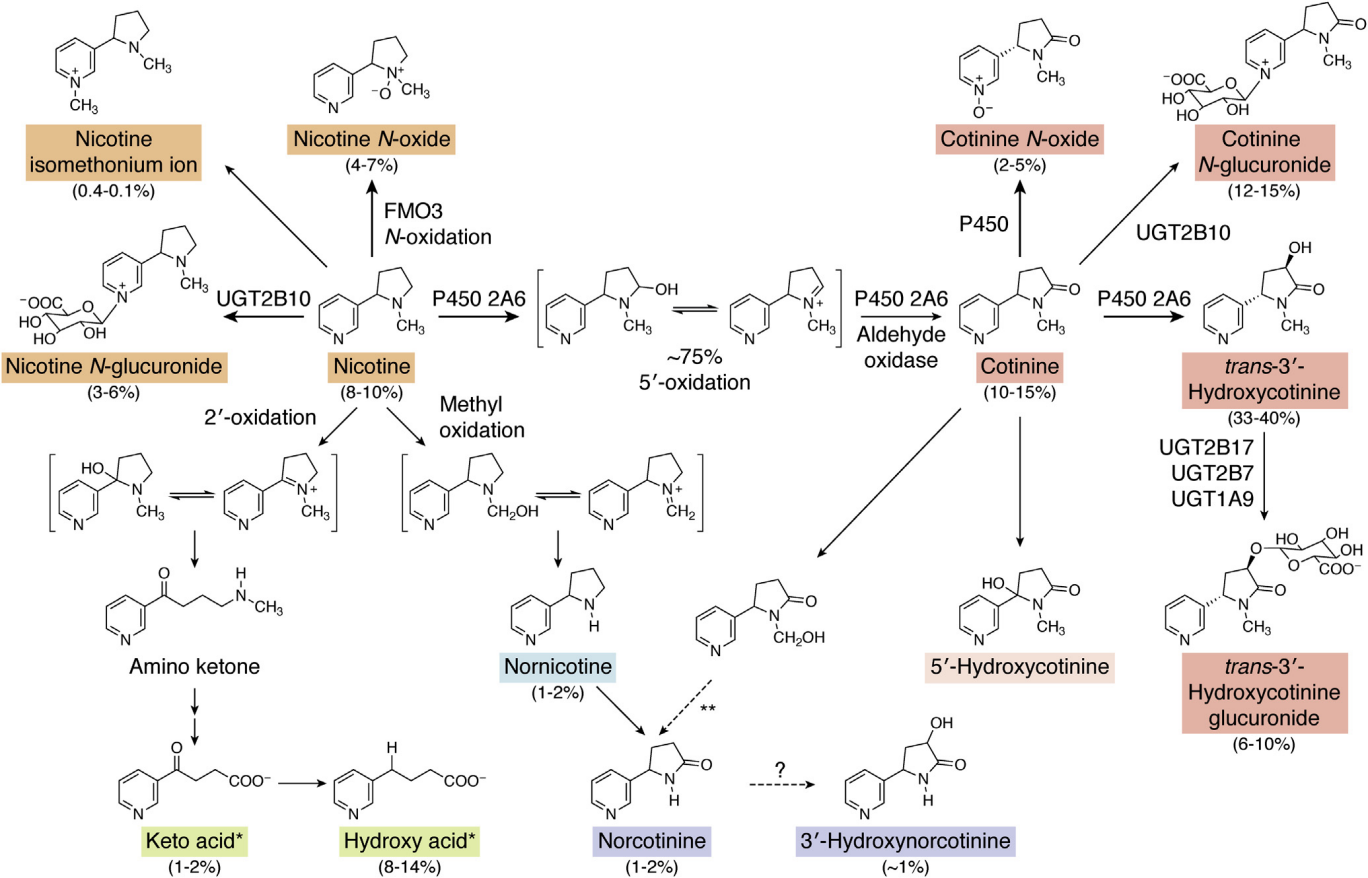
**Nicotine metabolism pathways in smokers.** The compounds with boxed names have been quantified as urinary metabolites in smokers. The percentages are estimated levels in smokers who are not deficient in P450 2A6 or UGT2B10 activity (32, 38, 39, 40). ∗4-hydroxy-4-(3-pyridyl)butanoic acid (hydroxy acid), 4-oxo-4-(3-pyridyl)butanoic acid (keto acid), 4-(methylamino)-1-(3-pyridyl)-1-butanone (aminoketone); ∗∗Norcotinine is a product of P450 2A6–catalyzed cotinine metabolism, but norcotinine was not present in the urine of individuals who were administered cotinine.

The next phase of the study of nicotine metabolism, the characterization of important enzyme catalysts, brought biochemists and pharmacologists into the field. These investigations began with microsomal studies in the 1970s and 80s (19, 20, 21, 22). Also, in the 1980s, the addictive nature of nicotine became well recognized (8). This knowledge, combined with the realization that most smokers metabolize more than 80% of the nicotine they consume to cotinine, led to the hypothesis that the activity of the enzyme responsible for nicotine 5′-oxidation would influence smoking behaviors (23). Early studies confirmed that the catalyst was a cytochrome P450 (P450, CYP) enzyme (19, 22) but not until 1996 was P450 2A6 identified as the primary human enzyme responsible for nicotine 5′-oxidation (24, 25). This discovery resulted in numerous studies that investigated the relationship of genetic variants of *CYP2A6* to smoking behavior (26). A number of these have confirmed an association between *CYP2A6* genotype and cigarettes per day.

The relative risk of lung cancer is significantly influenced by smoking dose, and *CYP2A6* variants that affect smoking would be expected to influence the risk. Many, but not all epidemiological studies have reported an association between *CYP2A6* variants and the risk of a smoker to develop lung cancer (27). The quantitation of nicotine metabolites as biomarkers of tobacco exposure in lung cancer cases has confirmed that the relationship of *CYP2A6* genotype to lung cancer risk is mediated by the effect of variation in nicotine metabolism on smoking dose, as illustrated in Figure 2. The first studies to demonstrate this were carried out in populations with a high prevalence of *CYP2A6* nonfunctional variants, but now there is good evidence that *CYP2A6* genotype contributes to a smoker's lung cancer risk in a number of different populations.

**Figure 2:**
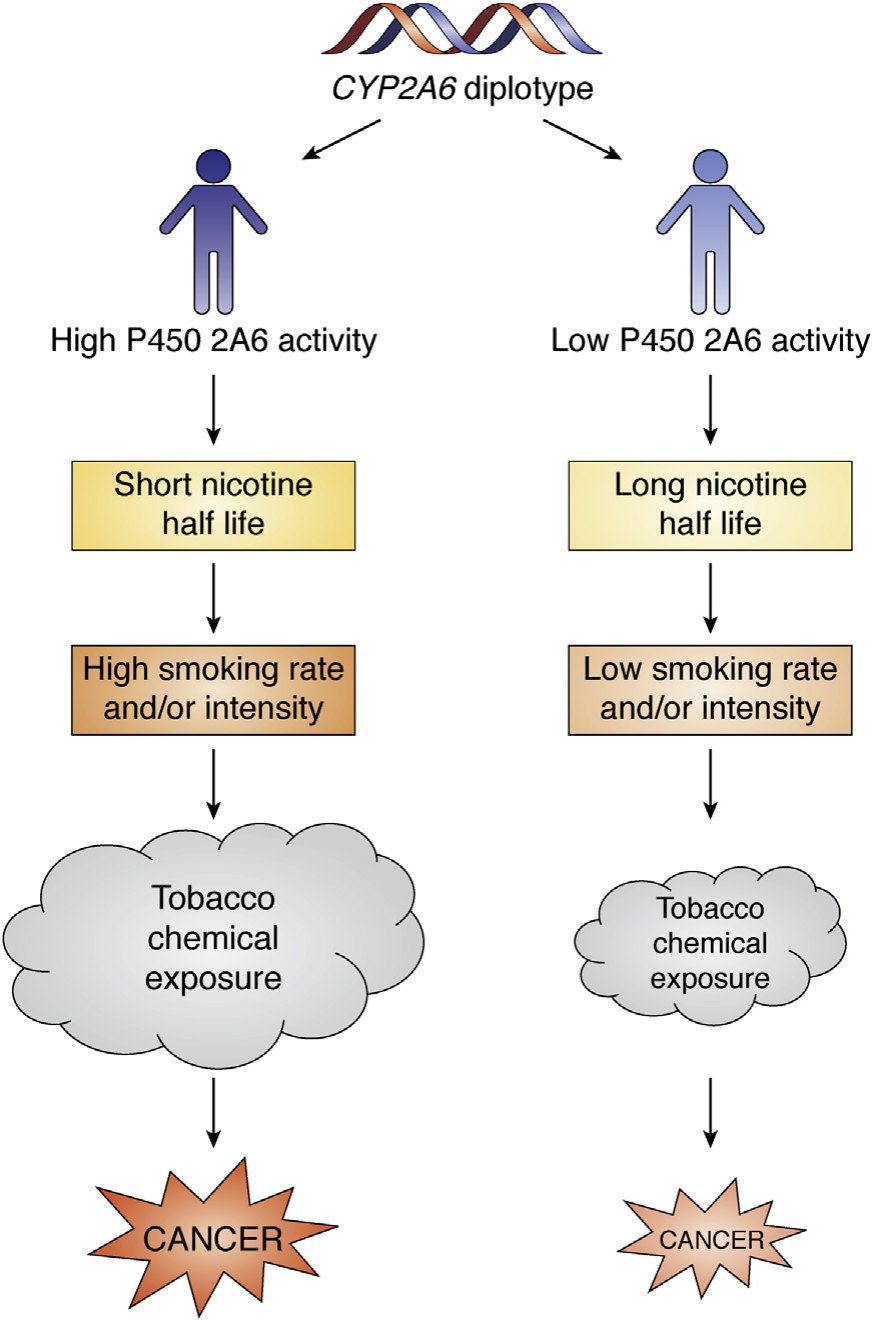
**Proposed relationship of *CYP2A6* diplotype to smoking intensity and cancer**.

There are more than 40 million smokers in the United States (28) and more than a billion worldwide (https://www.who.int/tobacco/global_report/2017/en/). Accurate measures of tobacco smoke exposure could improve lung cancer risk prediction and target smoking cessation interventions to those most at risk. In the United States today, more than 60% of lung cancers occur in former smokers (1). These individuals remain at an elevated risk of lung cancer more than 20 years after quitting, therefore identifying those at the greatest risk is critical to appropriately target lung cancer screening efforts (29). Current recommendations for screening are based on age and smoking history, measured in pack years (the number of years smoked times reported cigarettes per day). Cigarettes per day is an imprecise measure of smoking dose exposure (30, 31). In studies presented here on the relationship of nicotine metabolism to lung cancer, we illustrate how biomarkers of nicotine metabolism and tobacco exposure provide significantly better measures of smoking. These biomarkers have been key to elucidating our understanding of some of the observed racial/ethnic differences in lung cancer risk. The challenge now is to apply our knowledge of nicotine metabolism and smoking dose biomarkers to the lung cancer risk of former smokers. One approach is to develop a genetic model of P450 2A6-mediated nicotine metabolism to better predict smoking exposure in Former smokers. The predicted smoking dose derived from this model could be used to improve current lung cancer risk prediction models to identify former smokers at the highest risk of developing lung cancer.

The goal of this review is to highlight the metabolic pathways and nicotine-related biomarkers that are critical to understanding the contribution of nicotine metabolism to the etiology of lung cancer in smokers. The most recent comprehensive review of nicotine metabolism was published 15 years ago by Hukkanen *et al.* (32). Key findings since then, including the identification of the uridine 5′-diphospho-glucuronosyltransferase (UGT), 2B10 as the catalyst of nicotine and cotinine *N*-glucuronidation, *in vitro* studies of P450 2A6–catalyzed nicotine, nicotine ^Δ1′^^,5′^-iminium ion, and cotinine metabolism, and variation in the relative abundance of nicotine metabolic pathways in different racial/ethnic groups, will also be discussed. A more complete characterization of nicotine metabolic pathways by racial/ethnic group has contributed to our understanding of the variable incidence of lung cancer in smokers from these groups.

## Overview of nicotine metabolism

Two nicotine metabolism pathways are common to all mammals, 5′-oxidation, and *N*-oxidation. Studies have been carried out in many mammalian systems, from humans to mice (22, 32, 33, 34, 35), and more than 20 nicotine metabolites have been identified (Fig. 1). Several comprehensive reviews are available that discuss much of this work (21, 22, 32, 36, 37).

In humans, nicotine is metabolized by three primary pathways: P450-catalyzed 5′-oxidation, UGT-catalyzed *N*-glucuronidation, and flavin monooxygenase (FMO)–catalyzed *N*′-oxidation (Fig. 1). The ^Δ1′^^,5′^-iminium ion product of nicotine 5′-oxidation is further metabolized to cotinine. The formation of cotinine is quantitatively the most important nicotine metabolism pathway (32). Three minor pathways: methylation of the pyridine nitrogen to the nicotine isomethonium ion, 2′-oxidation, and oxidative *N*-demethylation also contribute to nicotine metabolism.

Cotinine, like nicotine is metabolized by three major pathways: 3′-oxidation to *trans* 3′-hydroxycotinine, cotinine *N*-glucuronidation, and cotinine *N*-oxidation (Fig. 1). In contrast to nicotine, the *N*-oxidation of cotinine occurs on the pyrrolidine nitrogen not the pyridine nitrogen, and the catalyst of this reaction is a P450 enzyme not an FMO (32). 3′-Hydroxycotinine is further metabolized to its O-glucuronide conjugate. Minor metabolites of cotinine include 5′-hydroxycotinine and norcotinine.

## Nicotine metabolites excreted by smokers

In addition to cotinine, 12 urinary nicotine metabolites have been identified (21, 32, 37, 38). The pathways that give rise to these metabolites are presented in Figure 1, and the names of the compounds are boxed. A 14th possible urinary metabolite, 5′-hydroxycotinine, is shown; the name is in a dashed box since the concentration of this compound in urine has only been reported in a review article (37). The estimated percent of each metabolite in the urine of smokers who are not deficient in P450 2A6 or UGT2B10 are presented. These estimates are updated based on more recent data and slightly modified from those presented in the review by Hukkanen *et al.* (32, 38, 39, 40). The importance of P450 2A6 and UGT2B10, the predominant catalysts of nicotine and cotinine oxidation and *N*-glucuronidation, to overall nicotine metabolism is discussed later in separate sections.

In smokers, eight metabolites (nicotine *N*-oxide, nicotine glucuronide, cotinine, cotinine glucuronide, cotinine *N*-oxide, 3′-hydroxycotinine, 3′-hydroxycotinine glucuronide, and 4-hydroxy-4-(3-pyridyl)butanoic acid (hydroxy acid)) plus unmetabolized nicotine account for >90% of the nicotine dose (18, 32, 40, 41). The other five nicotine metabolites that have been quantified each account for <1 or 2% of the nicotine metabolites excreted by a smoker (32, 38, 42). These minor metabolites are discussed briefly here. Nornicotine and norcotinine are both found in the urine of smokers, but norcotinine was not detected in the urine of individuals administered cotinine (43). However, Hukkanen *et al.* (32) reported in unpublished data that smokers administered D_4_-cotinine excreted D_4_-norcotinine. Also, norcotinine is a major product of P450 2A6–catalyzed cotinine metabolism *in vitro* (44). Norcotinine is found in the urine of dogs administered nornicotine, and nornicotine is a very minor metabolite of P450 2A6–catalyzed nicotine metabolism (45, 46). It is unclear from these data if the norcotinine excreted by smokers is a product of cotinine or nornicotine metabolism; both pathways are presented in Figure 1. Nornicotine is present in tobacco smoke, and a portion of the nornicotine in smokers is from that exposure (18). The urinary concentration of the nicotine isomethonium ion was quantified in smoker's urine by Neurath *et al.* (16) but has rarely been measured by others. The most recently identified nicotine metabolite, 3′-hydroxynorcotinine (38), could be a product of norcotinine oxidation (Fig. 1), or it might form by the demethylation of 3′-hydroxycotinine. It is unknown if one or both pathways occur in smokers.

4-Oxo-4-(3-pyridyl)butanoic acid (keto acid), the precursor of hydroxy acid, is a minor urinary nicotine metabolite in smokers, but the sum of these two acids accounts for as much as 15% of the nicotine dose excreted (39, 40, 41, 47). In dogs and rats, keto acid and hydroxy acid are metabolites of cotinine and are proposed to form from 5′-hydroxycotinine or norcotinine (32, 48), but neither norcotinine nor hydroxy acid has been detected in humans administered cotinine (43, 48, 49). Human liver microsomal metabolism of nicotine by 2′-oxidation generates keto acid (49); therefore, this pathway of keto acid and hydroxy acid formation is illustrated in Figure 1.

**Figure 3:**
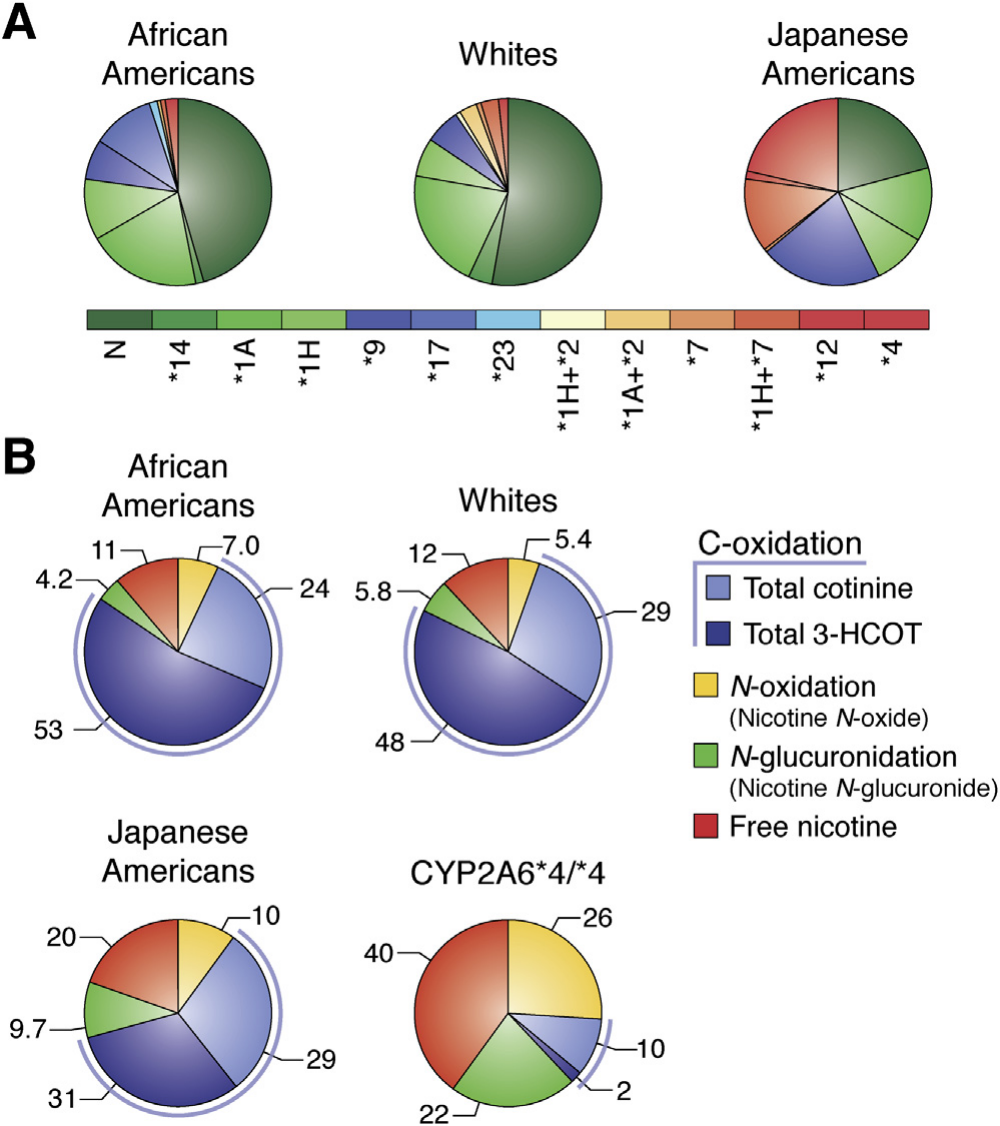
**The distribution of *CYP2A6* haplotypes and urinary nicotine metabolites in African American, White, and Japanese American smokers in the multiethnic cohort.***A*, *CYP2A6* haplotypes described by eight single nucleotide polymorphisms and two copy number variants (∗12 and ∗4) and listed in the order of predicted metabolic activity (normal [*green*] to nonfunctional or deleted [*red*]). The allele nomenclature is as described at https://www.pharmvar.org/htdocs/archive/cyp2a6.htm. Figure copyright © 2016, Oxford University Press, reused with permission (52). *B*, the proportion of nicotine metabolized by C-oxidation, *N*-glucuronidation, and *N*-oxidation in three racial/ethnic groups. The values for each slice of the pie are the mean percentage of the compounds excreted relative to total nicotine equivalents for African Americans (n = 364), Whites (n = 437), Japanese Americans (n = 674), and smokers homozygous for the *CYP2A6 ∗4* allele (n = 34) who are all Japanese American (50, 52). The figure is modified from the study by Murphy *et al.*, 2014 (50).

In smokers not deficient in P450 2A6 activity, 75% to 80% of the nicotine dose is metabolized to cotinine and its metabolites (18, 32, 50). However, each smoker's urinary nicotine metabolite profile depends on the relative abundance and activity of the enzymes involved. Significant differences in the relative frequency of genetic variants of P450 2A6 (gene *CYP2A6*) across racial/ethnic groups (Fig. 3*A* and Table 1) result in variation in the urinary nicotine metabolite profile (Fig. 3*B*) of smokers from these groups (50). Smokers of Japanese ancestry have a high frequency of low or no activity of *CYP2A6* alleles, and this is reflected in the reduced proportion of nicotine metabolized by 5′-oxidation. In individuals who are homozygous for *CYP2A6*∗4, a deletion allele (Fig. 3*B*), nicotine 5′-oxidation is a minor pathway, and the percentage of nicotine excreted unchanged increases, as does nicotine metabolism by *N*-glucuronidation and/or *N*-oxidation (50, 51, 52).

**Table 1:** *CYP2A6* variants with an allele frequency greater than 1% in one of five populations

Allele	Defining variant	Variant type	Functional consequence (*in vivo* unless noted)	Allele frequency in various populations (%)^a^				
				EUR	AFR	EAS	SAS	AMR
∗1	None			64.6	65.1	**30.8**	65.6	71.9
∗1A	rs1137115	51 G>A, synonymous	Reduced mRNA expression and reduced activity	20	20	13	NA	14
∗1B		58 bp gene conversion 3′ UTR	Increased mRNA stability	30	15	30	NA	NA
∗1H	rs61663607	−745 A>G	lower mRNA expression *in vitro*	11	8	**21**	NA	4
∗2	rs1801272	Missense (L160H)	No activity	**2.3**	0.5	0	1.1	1.2
∗4	Whole gene deletion		No activity	1	1.5	**17**	7	4
∗7	rs5031016	Missense I471T	Much reduced activity	<0.1	<0.1	**12.9**	0.3	0.3
∗9	rs28399468	Disrupts TATA box	Reduced activity	11.1	8.3	**23**	14.4	13.8
∗12	Hybrid allele, exon 1 and 2 from CYP2A7 (52, 121, 165)		No activity	1–3	<1	1.4	NA	3
∗14	rs28399435, rs1137115	Missense (S29N), 51 G>A	No change activity	1.4	0.8	<0.1	3.5	1.2
∗17	rs28399454	Missense (V365M)	Reduced activity	0	**11.2**	0	0	0.6
∗18	rs1809810	Missense (Y392F)	Reduced activity *in vitro*	1.5	0.6	1.2	1.4	1.1
∗19	rs5031016, rs1809010	Missense (I471T, Y392F)	Reduced activity	<0.1	<0.1	1.2	0.3	0.3
∗20	Frameshift exon 4		Truncated protein, little activity	0	**1.5**	0	NA	NA
∗21	rs6413474	Missense (K476R)	Reduced *in vitro*	**2.8**	0.2	<0.1	1.9	0.3
∗23	rs56256500	Missense (R203C)	Reduced activity	0		0	<0.1	<0.1
∗25	rs28399440	Missense (F118L)	Reduced activity	0	**1.4**	0	0	0
∗28	rs28399440, rs8192730	Missense (N418D, E419D)	Reduced activity	0	**2.0**	0.1	<0.1	0.2
∗35	rs143731390 (rs61736436)^b^	Missense (N438Y)	Reduced activity	14.9	5.9	12.8	3.7	4.5
∗1X2	Whole gene duplication		Increased metabolism	~1%	0	<0.5	NA	NA

NA, not available.

a Data presented are from Zhou *et al.*, 2017 (122) who derived the frequencies from exome sequencing data of 56,945 provided by the Exome Aggregate Consortium, except data for ∗1A and ∗1H, which are from the studies of Bloom *et al.*, 2012 (165) and Park *et al.*, 2016 (52). ∗1B and ∗1X2 are from the study of Tanner *et al.*, 2017 (121) and ∗12 references (52, 121, 165). Values are bolded when that group has a more than twofold greater frequency than any other group. The groups are Europeans (EUR), Africans (AFR), East Asians (EAS), South Asians (SAS), and admixed Americans (AMR). Frequencies for CYP2A6∗1 do not consider variants of ∗1A, ∗1B, and ∗1H.

b Earlier publications refer to this rs number (123, 165); the frequencies reported by Zhou *et al.*, 2017 (122) are much different than those reported by Tanner *et al.*, 2017 (121).

## Nicotine metabolism to cotinine and *trans* 3′-hydroxycotinine

When nicotine is inhaled, vaped, absorbed from a patch, or swallowed from smokeless tobacco or nicotine gum, it is metabolized relatively quickly to cotinine (32, 53). Metabolism occurs predominantly in the liver (32). In human liver microsomes, P450 2A6 is the key catalyst in the transformation of nicotine to cotinine (24, 25, 54). P450 2A6 catalyzes the 5′-oxidation of nicotine to the ^Δ1′^^,5′^-iminium ion, which is then oxidized to cotinine (Fig. 1). The oxidation of the iminium ion may be catalyzed by a cytosolic aldehyde oxidase or by P450 2A6 (46, 55, 56, 57). In the study of microsomal metabolism of nicotine, cytosol is often added as a source of aldehyde oxidase. However, human liver microsomes and P450 2A6 convert nicotine to cotinine in the absence of cytosol (46, 58), and P450 2A6 catalyzes the oxidation of the ^Δ1′^^,5′^-iminium ion to cotinine (57). Therefore, aldehyde oxidase is not required to metabolize nicotine to cotinine *in vitro* and may not be necessary *in vivo*. P450 2B6 is the only other human enzyme that catalyzes the 5′-oxidation of nicotine, but it is a much less efficient enzyme than P450 2A6 (58, 59, 60). Interestingly, P450 2B6 does not convert nicotine to cotinine in the absence of cytosol (58). That is, P450 2B6, unlike P450 2A6, does not catalyze the oxidation of the Δ^5′^^(1′^^)^-iminium ion. *In vitro*, both enzymes catalyze a small amount of nicotine demethylation (58, 61). When P450 2A6 is present, P450 2B6 appears to contribute little to hepatic nicotine metabolism, either 5′-oxidation or *N*-demethylation (58, 61).

The metabolism of cotinine to 3′-hydroxycotinine is also catalyzed by P450 2A6, although less efficiently than the 5′-oxidation of nicotine (44, 62). No other human P450 has been identified as a catalyst of this reaction, and little or no 3′-hydroxycotinine is excreted as a nicotine metabolite by individuals who do not express P450 2A6 (Fig. 3*B*) (63). Therefore, the ratio of 3′-hydroxycotinine to cotinine (in urine, plasma, and saliva) has been characterized as a measure of P450 2A6 activity in smokers and is referred to as the nicotine metabolism ratio (32, 64, 65).

## P450 2A6–catalyzed metabolism of nicotine, Δ^1′^^(^^5′^^)^iminium ion, and cotinine *in vitro*

P450 2A6–catalyzed nicotine metabolism has typically been quantified by measuring the formation of cotinine in the presence of aldehyde oxidase (25, 49, 54, 59). However, the nicotine ^Δ1′^^,5′^-iminium ion has been quantified directly as the major product of P450 2A6–catalyzed nicotine metabolism in both heterologous expression systems and with human liver microsomes (46, 66). Additional metabolites were also identified. The products of methyl oxidation and 2′-oxidation, 4-(methylamino)-1-(3-pyridyl)-1-butanone aminoketone, and nornicotine were minor metabolites of P450 2A6–catalyzed metabolism (Fig. 1) (46). Aminoketone was also a minor metabolite of human liver microsomal nicotine metabolism (46, 49). The amount of nicotine 2′-oxidation relative to 5′-oxidation was from <2% to 11% in these reactions. The higher percentage of nicotine metabolism by 2′-oxidation was estimated from the ratio of amino ketone to cotinine formation in the presence of cytosol (49). However, based on later experiments (46), it is likely that in the original experiment, not all the iminium ions were converted to cotinine. Therefore, total 5′-oxidation accounted for a higher percentage of nicotine metabolism than what was reported (49), and the relative amount of 2′-oxidation was closer to 1 to 2%. In the earlier publication, a relatively large peak at the correct retention time of nicotine ^Δ1′^^,5′^-iminium ion was observed in the HPLC chromatograms from which aminoketone and cotinine were quantified. The overwhelming predominance of 5′-oxidation relative to 2′-oxidation is supported by the X-ray structure of nicotine complexed with P450 2A6 (67). In this structure, nicotine is oriented with the 5′-pyrrolidine carbon within 4 Å of the heme; the 2′-carbon is much further away.

The oxidation of nicotine ^Δ1′^^,5′^-iminium ion to cotinine by a P450 enzyme was first observed in experiments on nicotine metabolism with rabbit liver microsomes (68). In more recent experiments, cotinine was quantified as the major product of P450 2A6–catalyzed metabolism of the iminium ion (57). A minor product of this reaction was tentatively identified as 2′-hydroxy-3′,4′-dehydronicotine or a related isomer, which would dehydrate to β-nicotyrine. Interestingly, cotinine and cotinine metabolites were also detected as products of P450 2A6–catalyzed nicotine ^Δ1′^^,5′^-iminium ion, even at short incubation times. The cotinine metabolites detected were 3′-hydroxycotinine, norcotinine, and *N*-hydroxynorcotinine (Fig. 1). Additional experiments supported the hypothesis that the majority of the 3′-hydroxycotinine formed was the product of the sequential metabolism of the nicotine ^Δ1′^^,5′^-iminium ion (57).

The *in vitro* metabolism of nicotine and the iminium ion support the argument that the urinary products of nicotine 2′-oxidation, hydroxy acid, and keto acid, and the product of methyl oxidation, nornicotine, may depend on P450 2A6 activity in smokers (46, 49, 57). The relationship of P450 2A6 activity to the formation of these metabolites has not been investigated. Characterizing whether smokers who have no *CYP2A6* activity excrete hydroxy acid, which is a relatively abundant nicotine metabolite, would allow a more complete phenotyping of smokers for P450 2A6 activity.

The *in vitro* metabolism of cotinine by *CYP2A6* results in the formation of three products; the major product is *N*-(hydroxymethyl)norcotinine not 3′-hydroxycotinine (44). This is surprising given that in smokers norcotinine is a minor nicotine metabolite. One explanation for the discrepancy in the relative abundance of these two pathways *in vivo* and *in vitro* may be that a majority of the 3′-hydroxycotinine formed *in vivo* is from sequential metabolism of the nicotine ^Δ1′^^,5′^-iminium ion (57). That is, much of the cotinine product of the iminium ion does not leave the active site prior to further metabolism to 3′-hydroxycotinine.

## Nicotine *N*-oxide and FMO3

FMO3 is the catalyst of nicotine *N*-oxidation in smokers (32, 69). The primary product of this reaction *in vitro* is *trans* nicotine *N*-oxide, which is the only isomer detected in smoker's urine (69, 70). Genetic variants in FMO3 influence, albeit to a small extent, variance in the percentage of nicotine metabolized to cotinine (71). *In vitro* FMO1 is a similar or more efficient catalyst of nicotine *N*-oxidation than FMO3, but both *cis* and *trans* nicotine *N*-oxide are products of FMO1 metabolism (70, 72). FMO3 is primarily expressed in the liver, whereas FMO1 is expressed in extrahepatic tissues, including the brain (73). FMO1 does not contribute significantly to total nicotine *N*-oxidation in smokers, but it may contribute to nicotine metabolism in the brain. Human brain tissue has FMO activity, and microsomes prepared from human brain catalyze both *cis* and *trans* oxidation of nicotine but with different kinetic parameters (74, 75). These data support a role for both FMO1 and FMO3 in nicotine metabolism in the brain. Variation in the activity of these enzymes might influence nicotine addiction, by contributing to brain nicotine concentration and interaction with nicotinic cholinergic receptors. Interestingly, a genome-wide association study did report a significant association between single nucleotide polymorphisms in *FMO1* and nicotine dependence (70). In addition, polymorphisms in FMO3 were associated with cigarettes per day and nicotine dependence (71, 75).

## Glucuronide conjugation of nicotine, cotinine, and 3-hydroxycotinine

Prior to the structural characterization of the quaternary *N*^*1*^-glucuronide of nicotine (76), several investigators quantified the presence of a nicotine glucuronide in smokers (17, 18, 32, 77) from the urinary nicotine concentrations before and after treatment with β-glucuronidase. Cotinine *N*-glucuronide has similarly been characterized and quantified in smokers (17, 18, 32, 77, 78). In 2007, two research groups independently identified UGT2B10 as a catalyst of nicotine and cotinine *N*-glucuronidation (79, 80). Prior to this discovery, UGT1A4, which also catalyzes these reactions, was believed to be the catalyst in smokers (32, 81, 82). However, UGT2B10 is a more efficient catalyst than is UGT1A4, and it is the enzyme responsible for nicotine and cotinine glucuronidation in human liver microsomes and in smokers (50, 79, 80, 83).

**Figure 4:**
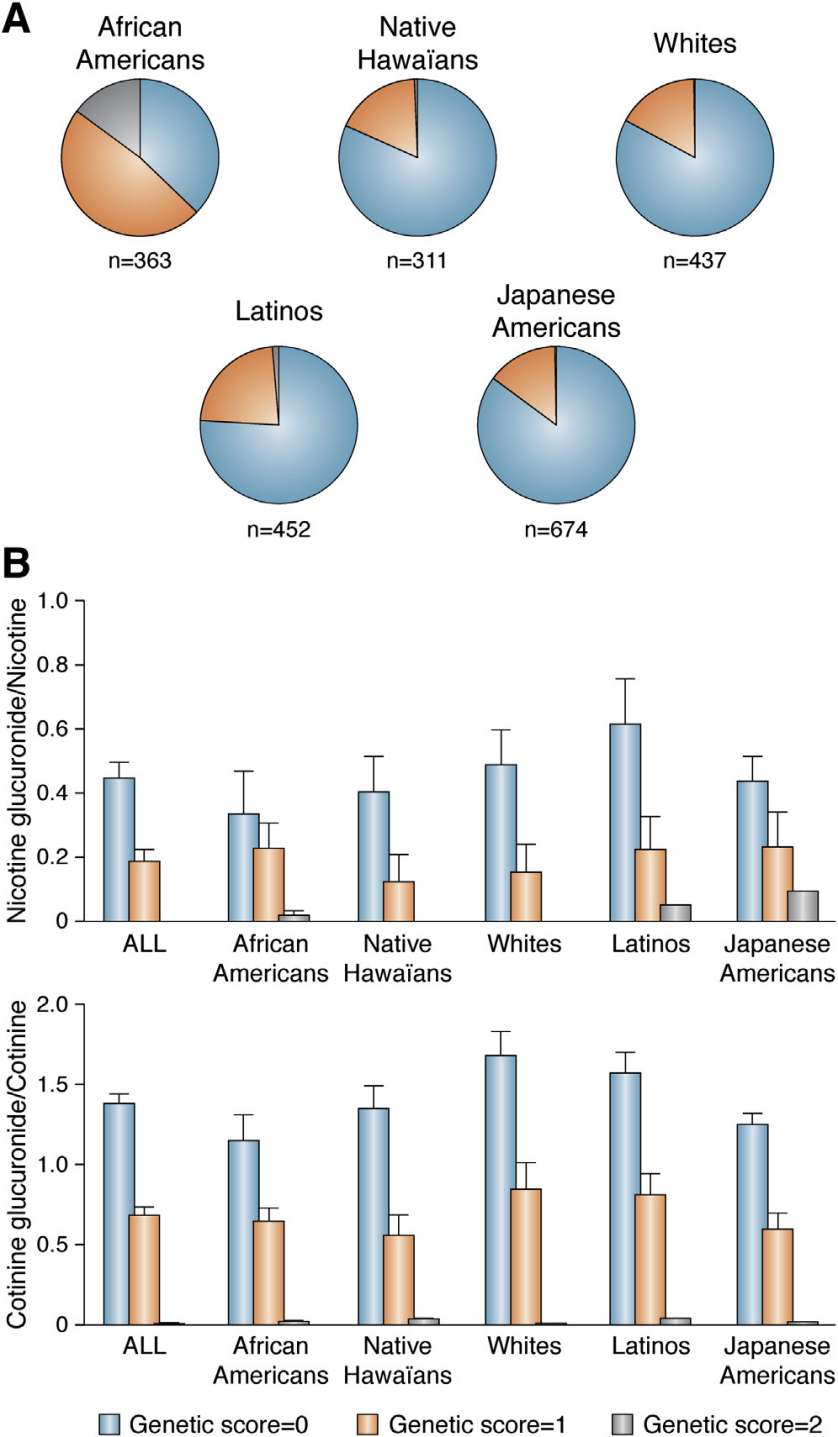
**The distribution of UGT2B10 variants and nicotine and cotinine glucuronidation phenotypes for the five racial/ethnic groups in the multiethnic cohort.***A*, the section of the pies represents the proportion of smokers with no (*blue*), one (*orange*), or two (*gray*) UGT2B10 alleles that code for no functional enzyme as described by genetic scores 0, 1, and 2. Genetic score 0, rs61750900 GG and rs2942857 AA; score 1, rs61750900 GT or rs2942857 CA; score 2, rs61750900 TT or rs2942857 CC, or both rs61750900 GT and rs2942857 CA. *B*, the urinary ratio of cotinine glucuronide to cotinine and nicotine glucuronide to nicotine excreted by smokers by UGT2B10 genetic score. The values, geometric means, and 95% confidence interval are adjusted for age, sex, creatinine, body mass index, and race. When n <10 for score 2, whites (n = 1), Japanese American (n = 2), Hawaiian (n = 2), and Latinos (n = 7), arithmetic means are presented and no confidence interval. The data used to create Figures A and B are from Table S3 of Murphy *et al.*, 2014 (50).

Two UGT2B10 variants that code for nonfunctional enzyme have been characterized (50, 79). Both significantly impact the extent of nicotine and cotinine glucuronidation in smokers (50, 83, 84, 85). White smokers homozygous for an Asp67Tyr UGT2B10 variant (rs6175900) excreted little if any cotinine or nicotine glucuronide (85), whereas heterozygous white smokers excreted about half as much of each glucuronide (84, 85). However, this variant, with a frequency of ∼10% in white *versus* ∼5% in African Americans, does not account for the significantly lower nicotine and cotinine glucuronidation in African American compared with white smokers reported more than 20 years ago (83, 86). A UGT2B10 splice variant (rs2942857) has now been identified that explains the decreased nicotine and cotinine glucuronidation in African American smokers (50, 87, 88). The frequency of the splice variant is 37% in individuals of African ancestry compared with <1% of whites (50, 87, 88, 89). About 30% of African Americans carry neither the splice nor the Asp67Tyr variant compared with >80% of whites (Fig. 4*A*; genetic score 0) (50, 90). Almost 20% of African Americans have no functional UGT2B10 enzyme (Fig. 4*A*; genetic score 2). Among smokers from five different racial/ethnic groups those who are heterozygous for either the splice or the Asp67Tyr variant excreted 50% less nicotine and cotinine glucuronide than individuals who carried neither variant (Fig. 4*B*; genetic score 1 *versus* 0) (50). Smokers homozygous for either variant (genetic score 2), more than 80% of whom are African American, excreted little or no nicotine or cotinine *N*-glucuronide. Interestingly, the relative glucuronidation of cotinine in African Americans carrying one or neither variant alleles is significantly lower (*p* < 0.05) than in whites with these genotypes (Fig. 4), possibly because of the presence of other UGT2B10 variants in African Americans that have yet to be characterized (50).

The glucuronide conjugate of 3′-hydroxycotinine excreted by smokers is the *O*-glucuronide. No 3′-hydroxycotinine *N*-glucuronide is found in the urine of smokers even though human liver microsomes catalyze both *O*- and *N*-glucuronidation of 3-HCOT (32, 91). UGT1A9, UGT2B7, and UGT2B17 are all catalysts of 3′-hydroxycotinine *O*-glucuronidation (92, 93). In smokers, UGT2B17 contributes significantly to the glucuronidation of 3′-hydroxycotinine. Individuals who are homozygous for a UGT2B17 deletion allele excrete much less 3HCOT glucuronide than do individuals who carry two functional alleles (50, 92). The frequency of the UGT2B17 deletion is from 75 to 85% in Asian populations (94). Either UGT1A9 or UGT2B7 must contribute to the remaining glucuronidation of 3′-hydroxycotinine that occurs in these smokers.

## Nicotine metabolites as biomarkers of tobacco exposure

Cigarettes are designed to efficiently deliver nicotine to the user. Therefore, nicotine itself or its metabolites are arguably the most reliable biomarkers for smoking exposure (53). Nicotine has a relatively short half-life (1–2 h). Whereas, cotinine has a half-life of 8 to 30 h (32), and the presence of cotinine in either blood or urine is widely used as a biomarker of tobacco exposure (95). Cotinine is the most abundant nicotine metabolite present in blood but typically accounts for <15% of the nicotine dose excreted in urine. The most commonly used urinary biomarker of tobacco exposure is total nicotine equivalents, which is the sum of the molar concentrations of nicotine and six metabolites.

Both blood and urine cotinine concentrations are affected by individual differences in nicotine and cotinine metabolism. One smoker who metabolizes either compound more or less efficiently than another smoker will likely have different levels of cotinine for the same nicotine dose. This is particularly important in the study of racial/ethnic differences in tobacco-related disease risk, since the frequencies of *UGT2B10* and *CYP2A6* alleles vary by racial/ethnic group (Figs. 3*A* and 4A). In addition, it has been recognized for many years that African Americans, who are active smokers or who are exposed to secondhand tobacco smoke, have significantly higher cotinine concentrations than whites with similar exposure (96, 97, 98, 99).

**Figure 5:**
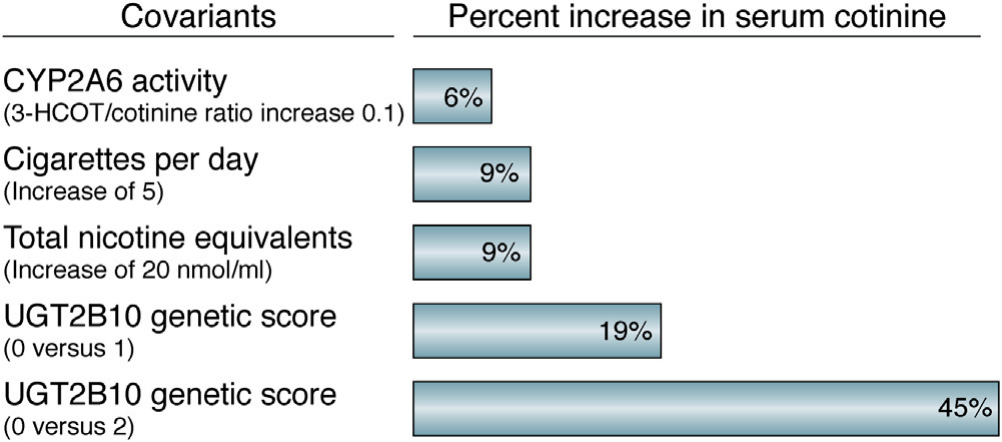
**Determinants of serum cotinine.** Values, adjusted for age, gender, and each of the other covariates, except UGT2B10 genetic score (0 *versus* 1), are the increase in the geometric mean cotinine concentration for the change in the covariant in a population of African American (n = 289) and non-Hispanic White smokers (n = 627). Genetic score 0, rs61750900 GG and rs2942857 AA; score 1, rs61750900 GT or rs2942857 CA; score 2, rs61750900 TT or rs2942857 CC, or both rs61750900 GT and rs2942857 CA. The data are from Table 3 and Table S2 in the study by Sipe *et al.* (101).

With the exception of smokers who carry two *CYP2A6* deleted alleles, UGT2B10 variants and cotinine glucuronidation levels influence blood cotinine concentrations significantly more than does P450 2A6 activity (100, 101). At similar levels of smoking, individuals who are homozygous for the *CYP2A6*∗4 deletion allele have significantly lower plasma cotinine levels than do individuals who carry *CYP2A6* alleles coding for fully functional enzymes (51, 63). However, in smokers with reduced P450 2A6 activity, the relationship of enzyme activity to plasma cotinine levels is complicated since both the formation of cotinine and its further metabolism is mediated by P450 2A6. Cotinine clearance, not formation, appears to dominate this process since plasma cotinine concentrations tend to be higher in individuals with detectable but reduced P450 2A6 activity (102). A significant amount of cotinine is metabolized by UGT2B10-catalyzed glucuronidation, and total cotinine clearance is 50% lower in individuals who are homozygous for the UGT2B10 splice variant compared with those who do not carry this allele (103). The decreased clearance of cotinine in these smokers results in 45% higher serum cotinine levels (101) (Fig. 5; genetic score 0 *versus* 2). Smokers who are heterozygous for either the UGT2B10 splice variant or the Asp67Tryr variant have on average 19% higher plasma cotinine concentrations. These differences are observed after adjustment for smoking dose (cigarettes per day and total nicotine equivalents) and P450 2A6 activity (101).

UGT2B10 genotype has a greater effect on plasma cotinine than does a significant increase in smoking does. An increase of five cigarettes per day or a total nicotine equivalent increase of 20 nmol/ml results in only a 9% change in mean plasma cotinine (Fig. 5). The UGT2B10 genotype effect is also greater than that observed for changes in P450 2A6 activity. A 0.1 change in the 3′-hydroxycotinine/cotinine ratio results in a 6% difference in plasma cotinine (Fig. 5). A 0.1 to 0.2 lower plasma ratio has been observed in smokers heterozygous for *CYP2A6* variant alleles ∗9, ∗2, and ∗4 (64, 104, 105).

Plasma cotinine concentrations are on average 50% higher in African Americans than in whites at the same levels of smoking, and much of this difference can be explained by the prevalence of UGT2B10 splice variant in African Americans (101). Recognizing the contribution of UGT2B10 genotype to serum (or saliva) cotinine levels is critical to understanding the relationship of an individual's cotinine level to smoking exposures and ultimately the relationship to lung cancer risk. This is true both in smokers and secondhand smoke–exposed individuals, since those with no UGT2B10 activity would appear to have a greater exposure based on cotinine levels.

UGT2B10 genotype also has a significant impact on urinary cotinine levels. UGT2B10-deficient smokers have significantly higher levels of urinary cotinine, whereas total cotinine levels, the sum of cotinine and cotinine glucuronide, are lower compared with smokers with “normal” UGT2B10 activity (50, 100, 101). Despite some limitations of cotinine as a biomarker of tobacco exposure, several studies have established plasma cotinine and urinary total cotinine levels as predictors of lung cancer risk after adjustment for cigarettes per day (106, 107, 108, 109, 110, 111, 112). However, these studies were carried out in populations exclusively or predominately of similar racial/ethnic ancestry, whites, or Chinese.

Total nicotine equivalents levels are a better biomarker of recent nicotine intake and tobacco exposure than is cotinine since the concentration is not influenced by nicotine metabolism (95, 113, 114). The benefit of total nicotine equivalents is that it is composed of the products of all the major nicotine metabolism pathways, nicotine *N*-oxide, nicotine *N*-glucuronide, cotinine, cotinine *N*-glucuronide, 3′-hydroxycotinine, and 3′-hydroxycotinine glucuronide (Fig. 1). The sum of these six compounds plus nicotine account for 85 to 90% of a smoker's nicotine dose (18, 32). Nicotine *N*-oxide is not always included in the measurement of total nicotine equivalents, and other more minor metabolites such as nornicotine, norcotinine, and cotinine *N*-oxide may be added (114, 115). Hydroxy acid, which makes up a significant proportion of the excreted nicotine dose (Fig. 1), is not currently included in total nicotine equivalents. The influence of variation in nicotine metabolism on the relative amount of hydroxy acid excreted by a smoker is unknown. As discussed previously, the most likely source of hydroxy acid in urine is as the product of nicotine 2′-oxidation. *In vitro*, this reaction is catalyzed by P450 2A6, albeit to a much lesser extent than 5′-oxidation. Other P450 enzymes, possibly P450 3A4, which catalyzes the 2′ hydroxylation of the structurally similar compound *N*′-nitrosonornicotine (116), may catalyze nicotine 2′-oxidation. It would be interesting to determine if the amount of hydroxy acid excretion is influenced by *CYP2A6* genotype.

Total nicotine equivalents and cotinine are not reliable biomarkers of tobacco exposure in individuals who use other nicotine-containing products, such as electronic (e) cigarettes and nicotine replacement therapies that are often used as a means to reduce tobacco use. Two biomarkers that can distinguish cigarette smoking from other sources of nicotine are NNAL, a metabolite of the tobacco-specific lung carcinogen 4-(methylnitrosamino-1-(3-pyridyl)-butanone) (NNK), and cyanoethyl mercapturic acid, a metabolite of acrylonitrile (117, 118). Acrylonitrile is a volatile toxicant found in substantial quantities in cigarette smoke. There is little human exposure to acrylonitrile, other than from tobacco, and cyanoethyl mercapturic acid has recently been validated as an excellent biomarker to distinguish e-cigarette use from smoking (118).

## Nicotine metabolism, smoking behavior, and *CYP2A6* variants

A smoker's rate of nicotine clearance contributes significantly to nicotine intake and in turn their smoking behavior; that is the number of cigarettes they smoke and how they smoke them (53). The relationship of nicotine clearance to nicotine intake was first quantitatively documented in a pharmacokinetic study of Chinese American smokers (119), and a few years later, the direct relationship of nicotine clearance to *CYP2A6* genotype was confirmed (64).

There are numerous *CYP2A6* variants, many of which significantly impact nicotine metabolism (120, 121, 122). The Human Cytochrome 450 Nomenclature database lists more than 75 different *CYP2A6* alleles (https://www.pharmvar.org/htdocs/archive/cyp2a6.htm). Variants with a minor allele frequency of >1% in any of the five major populations are listed in Table 1. These include single nucleotide changes, small deletions and insertions, gene deletions and duplications, and gene hybrids (123). Importantly, the prevalence of the individual alleles varies by racial/ethnic group. The deletion allele, *CYP2A6*∗4, is prevalent in East Asian populations, with an allele frequency of up to 20%. Other variants are found exclusively in one population, for example, CYP2A∗17 (allele frequency 11%) is found only in individuals of African ancestry and *CYP2A6*∗7 (allele frequency 13%) only in East Asians (52, 122).

The effects of the 11 amino acid substitutions present in *CYP2A6* variants (Table 1) on enzyme activity are not all well characterized. The L160H change in CYP2A6∗2 results in no functional enzyme since this variant does not incorporate heme (124). Several of the other amino acid substitutions affect the kinetic parameters of nicotine 5′-oxidation. Among these, the most significant impact is for I471T (CYP2A6∗7) and V365M (CYP2A6∗17), which have decreased intrinsic clearance of 83% and 69%, respectively (125, 126, 127, 128). Notably, the closely related P450 2A13 has a methionine residue at position 365, and this change contributes to the larger active site of P450 2A13 compared with P450 2A6 and influences the binding of nicotine (67). The R203C substitution in CYP2A6∗23 was reported to have similar activity to CYP2A6∗17; however, no kinetic parameters have been reported (129). The arginine at position 203 is present in a substrate recognition region of P450 2A6 (130). The amino acid changes in CYP2A6∗35 (N438Y) and CYP2A6∗18 (Y392F) have not been shown to significantly affect *k*_cat_/*K*_*m*_ values for nicotine 5′-oxidation, although modest changes in both *K*_*m*_ and *k*_cat_ have been reported for each (126, 128, 131). No change in the kinetic parameters for nicotine 5′-oxidation were reported for CYP2A6∗21 (K476R) (130). The rate of nicotine 5′-oxidation catalyzed by either CYP2A6∗28 and CYP2A6∗25 at 30 or 300 μM nicotine was no different than that of P450 2A6 (132). The kinetic parameters for these two variants have not been reported. The phenylalanine at position 118 is located in the highly conserved substrate recognition site-1 region of the CYP2A family, and mutations at this position, including F118L, affect the substrate specificity of flavonoids (130, 133, 134). Therefore, in most cases, *in vitro* nicotine metabolism by variant P450 2A6 enzymes parallels what has been observed *in vivo* (Table 1). However, it is important to recognize that an individual may carry multiple single nucleotide polymorphisms in *CYP2A6*, and each may differentially affect the catalytic activity as well as the expression of the enzyme.

Several studies have observed a relationship between cigarettes per day and *CYP2A6* genotype (135, 136, 137, 138). Many of these are in East Asian populations, which have a greater than 60% prevalence of functionally deficient *CYP2A6* alleles (Table 1) (122). *CYP2A6* genotype has also been shown to influence smoking intensity (mean and total puff volume) (139). Total nicotine equivalent levels are related to *CYP2A6* genotype (52, 140). Both total nicotine equivalents and cigarettes per day are influenced by a smoker's P450 2A6 activity and therefore by their ability to metabolize nicotine (52, 141). Several investigators are working to develop genetic risk models to predict the nicotine metabolism (142, 143). Ideally, these models could provide a better reflection of lifetime daily smoking than self-reported cigarettes per day or a single total nicotine equivalent measurement.

## Relationship of *CYP2A6* and nicotine metabolism to lung cancer

As discussed, variations in P450 2A6–catalyzed nicotine metabolism influence smoking intensity. P450 2A6 is also one of several catalysts of the bioactivation of the tobacco-specific lung carcinogen, NNK (58, 144, 145). Many studies, including the first to report an association between *CYP2A6* genotype and lung cancer (146), hypothesized that decreased activation of NNK in smokers with decreased P450 2A6 activity protects smokers from lung cancer risk. While this may be one mechanism by which *CYP2A6* genotype is associated with lung cancer risk, the direct relationship of NNK bioactivation to lung cancer risk has not been demonstrated. In contrast, much evidence has established a strong connection from *CYP2A6* genotype to nicotine metabolism to smoking dose and lung cancer risk (26, 140, 147). The significantly lower dose of carcinogens received by smokers who carry variant *CYP2A6* alleles (52), not decreased bioactivation of NNK, is likely the far greater contributor to their lower lung cancer risk.

A number of epidemiology studies have confirmed the protective effect of the *CYP2A6*∗4 deletion allele on a smoker's lung cancer risk (27, 135, 136, 146, 148, 149, 150). A meta-analysis of nine case control studies reported a more than 60% reduction in lung cancer incidence among subjects with no functional P450 2A6 (genotype *CYP2A6*∗4/∗4 or CYP2A∗2/∗2) compared with those who carry neither of these alleles (135, 136, 146, 148, 151, 152, 153, 154, 155). The lung cancer cases in most of these studies included a mixture of smokers and never smokers (typically defined as smoking <100 per lifetime) and, as would be expected, the significance of the protective effect was attenuated if it was not stratified by smoking status (149, 150). A few studies have found no relationship between *CYP2A6* genotype and lung cancer risk (151, 156, 157, 158). However, the majority, if not all, of these negative studies are due to the inclusion of never smokers or to an insufficient number of cases. The latter is a significant challenge, particularly in populations with relatively low frequencies of *CYP2A6* variant alleles. *CYP2A6* alleles that code for little or no active protein are relatively common in individuals of East Asian ancestry but are rare in whites (Table 1 and Fig. 3*A*).

A large early study in Japanese reported that subjects who carried one or more *CYP2A6* variant alleles (∗4, ∗7, ∗9, or ∗10) smoked significantly fewer cigarettes per day than those who carried none of these alleles (136). However, even after adjustment for cigarette consumption, the risk for lung cancer was significantly lower for smokers who carried the variant alleles. The authors suggested, as others have, that the additional effect of *CYP2A6* genotype on lung cancer risk was due to a reduction in P450 2A6–mediated bioactivation of NNK. A more likely explanation is that cigarettes per day is a poor measure of smoking dose, and that a smoker's *CYP2A6* genotype is “correcting” for differences in nicotine and carcinogen consumption per cigarette. That is, smokers who carry variant *CYP2A6* alleles may smoke each cigarette less intensely than other smokers, inhaling less often or less deeply. More recent studies that use nicotine and carcinogen biomarkers to measure smoking dose support this hypothesis (140, 147, 159, 160).

**Figure 6:**
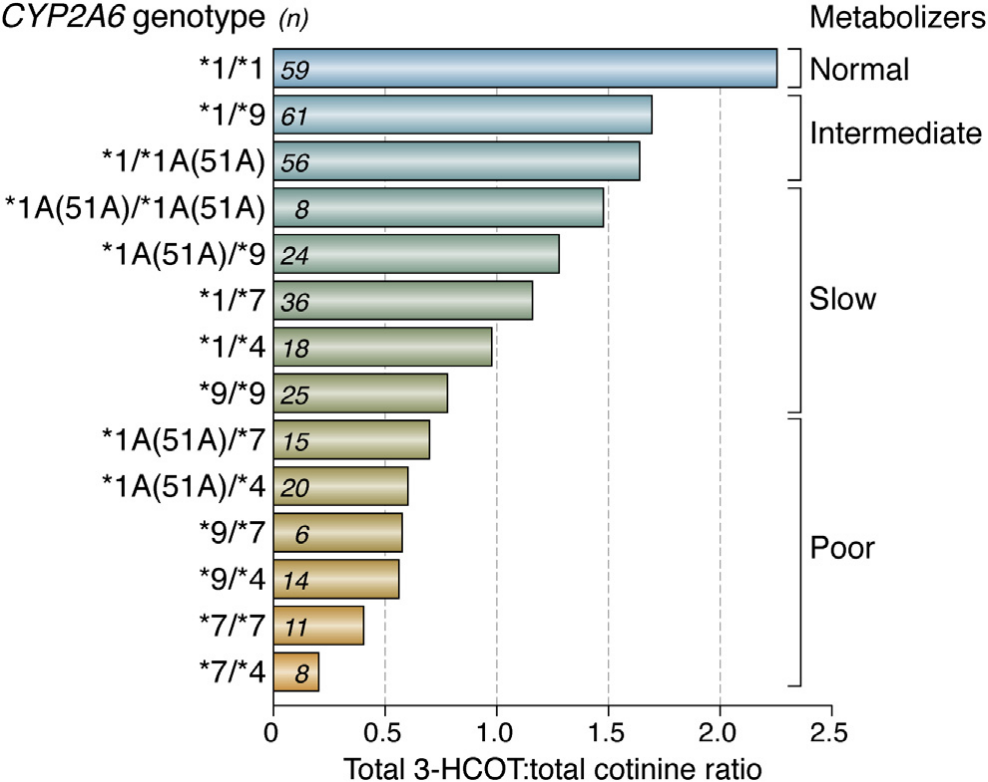
**Nicotine metabolism phenotype by *CYP2A6* diplotype in the Shanghai cohort.** The values are geometric means of the urinary total *trans*-3′-hydroxycotinine (3HC) to total cotinine ratio for smokers with different *CYP2A6* genotype that are grouped by metabolizer phenotype (140). Figure copyright © 2015, John Wiley and Sons, reused with permission.

In a study of Shanghai Chinese, the direct relationship of *CYP2A6* genotype to nicotine metabolism, smoking dose, and lung cancer risk was demonstrated (140). In this cohort, *CYP2A6* genotype predicted P450 2A6 activity, measured as the urinary ratio of 3′-hydroxycotinine to cotinine, and the risk of lung cancer was 30% lower for predicted poor metabolizers compared with other groups (Fig. 6). The protective effect of CYP2A6 genotype on lung cancer risk was no longer significant when adjusted for total nicotine equivalents, since nicotine consumption was mediating this association. In a study of similar design in Singapore Chinese, *CYP2A6* genotype was also associated with both total nicotine equivalent levels and a reduced risk of developing lung cancer (159).

Only a handful of studies in non-Asian populations have found a significant relationship between *CYP2A6* genotype and lung cancer risk (138, 153, 161). In a large population-based study of just over 3000 smokers, *CYP2A6*∗2 genotype, which codes for nonfunctional enzyme, was inversely associated with lung cancer risk (153). In a case control study in light smokers of European ancestry, fewer lung cancer cases carried one or more *CYP2A6* variant (∗2, ∗4, ∗9, or ∗12), than did controls (138). *CYP2A6* genotype was also associated with lung cancer in two African American cohorts (161). Male smokers in these cohorts with one or more copies of *CYP2A6*∗2, ∗4, ∗9, ∗17, ∗25, ∗26, ∗27, ∗31, or ∗35 (Table 1) were less likely to have lung cancer compared with those who carried none of these alleles.

The observed variation in lung cancer risk by racial/ethnic group (6, 7, 162) is based on reported cigarettes per day. However, cigarettes per day is a crude and often poor measure of tobacco exposure (30, 31). To address this limitation, total nicotine equivalents and biomarkers for several tobacco-related carcinogens (NNK, benzene, and butadiene) were measured in urine from a subset of ~2000 smokers in a large multiethnic prospective lung cancer study (6, 50). The urine was collected many years prior to a diagnosis of lung cancer. The concentrations of these biomarkers paralleled lung cancer risk in African Americans, white, and Japanese American smokers. Among the 2000 smokers for whom biomarkers were analyzed, 92 lung cancer cases were prospectively identified. The cases were distributed relatively equally across the racial/ethnic groups. After adjustment for age, sex, race/ethnicity, body mass index, and smoking duration, both P450 2A6 activity (the urinary ratio of total 3′-hydroxycotinine to cotinine) and total nicotine equivalents were associated with an increase in lung cancer risk (*p* value = 0.002). The association for P450 2A6 activity remained even after adjusting for cigarettes per day and total nicotine equivalents. These findings suggest that P450 2A6 activity provides information on lung cancer risk that is not captured by smoking history or total nicotine equivalents. It may be that P450 2A6 activity is a better predictor of lifetime smoking levels than is a single total nicotine equivalent measurement or reported cigarettes per day, and therefore an important variable to include in lung cancer prediction models.

A large collaborative study provided strong evidence for the contribution of *CYP2A6*-mediated nicotine metabolism to lung cancer risk in a non-Asian population (160). The investigators found that several single nucleotide polymorphisms associated with P450 2A6 activity and total nicotine equivalents in 2000 current smokers were also associated with lung cancer at the genome-wide significance level in over 13,000 cases of European descent.

Notably, in the recent update of lung cancer in the Multiethnic Cohort when predicted total nicotine equivalents replaced cigarettes per day as the measure of smoking, the relative risk for lung cancer among Japanese Americans, African Americans, and whites no longer differed (7). Predicted total nicotine equivalents were based on levels in the subset of 2000 smokers on whom this measure was available. For smokers of Japanese ancestry, total nicotine equivalent levels are driven by the prevalence of CYPA6 variants and smoking intensity (52). Smokers of African American ancestry with *CYP2A6* low-activity variants also have lower total nicotine equivalent levels (52). However, at the population level, other factors that contribute to the higher average total nicotine equivalent levels observed in African Americans mask the effect of *CYP2A6* genotype. These factors may be genetic or environmental and may include variants of the nicotine receptor (138) or socioeconomic differences (163) that influence smoking behavior (164).

Taken together, these lung cancer studies of *CYP2A6* genotype, P450 2A6 metabolism, and total nicotine equivalents confirm the relationship of *CYP2A6* genotype to lung cancer risk in individual smokers independent of their racial/ethnic identity (Fig. 2). At the population level, the relationship is most easily observed in those groups with a significant prevalence of variant alleles relative to other factors that affect smoking dose. This review is focused on the role of nicotine metabolism in lung cancer, but the risk of any tobacco-related disease, such as heart disease, chronic obstructive pulmonary disease, and other cancers that increases with smoking dose would likely be associated with P450 2A6-mediated nicotine metabolism.

## Conclusion

This review provides a comprehensive overview of nicotine metabolism; a summary of the use of biomarkers to define smoking dose; and an overview of molecular epidemiology studies of *CYP2A6* genotype, nicotine metabolism, and the risk of lung cancer across different racial/ethnic groups. The discussion of nicotine metabolism is focused on new findings published since 2005 on metabolites and enzyme catalysts, including P450 2A6, UGT2B10, and to a lesser extent FMOs. Nicotine is not a carcinogen, but it is the critical constituent of tobacco that drives continued smoking. The lung cancer studies described are presented to delineate the importance of *CYP2A6* to nicotine and tobacco exposure. Together these very different avenues of research have furthered our understanding of the relative contribution of P450 2A6 activity to an individual smoker's lung cancer risk.

One recent finding of note is the recognition that a *UGT2B10* splice variant is a significant contributor to the higher plasma cotinine levels found in smokers of African compared with European ancestry (100, 101). This difference in cotinine was first reported more than 30 years ago, and it is, in part because of more intense smoking (98). We now know that UGT2B10 genotype is a much greater determinant of plasma cotinine than is nicotine dose (Fig. 5). Why does this matter? *UGT2B10* genotype is not associated with lung cancer risk. But it influences a widely used biomarker of tobacco exposure. Smoking intensity is an important contributor to the greater lung cancer risk of African American relative to white smokers (6, 7). However, plasma cotinine is not an appropriate biomarker to compare smoking levels across these two groups; total nicotine equivalents are much better. This is one example of how a comprehensive understanding of nicotine metabolism enzymes, and the variants of those enzymes, is critical to the characterization of the variables contributing to a smoker's lung cancer risk. Future studies will continue to refine our understanding of how genetic variants influence individual smoker's nicotine metabolic profile.

*CYP2A6* genotype is associated with lung cancer risk in smokers. In racial/ethnic groups with a relatively high prevalence of *CYP2A6* variants, this association contributes significantly to the population-level risk of disease. In other populations, the contribution is much less. *CYP2A6* is highly polymorphic, and the frequency of variant alleles differs significantly by racial/ethnic group (Table 1). To appropriately weigh the role of *CYP2A6* variants on an individual's lung cancer risk will require the development of genetic models of nicotine metabolism that predict smoking dose. To develop a measure of smoking that is better than reported cigarettes per day, these *CYP2A6*-based models will need to be augmented with other variables, such as genetic variants of nicotinic receptors that mediate nicotine addiction. This predicted measure of smoking dose could then be used to access the lung cancer risk of the many former smokers who go on to develop lung cancer years after quitting. Ideally, this model of smoking dose could be combined with other lung cancer risk variables to more accurately identify former smokers at the highest risk and to intervene accordingly.

## Conflict of interest

The author declares that she has no conflicts of interest with the contents of this article.
